# The association between the dietary index for gut microbiota and its components with cardiovascular disease risk: a cross-sectional study based on NHANES

**DOI:** 10.3389/fnut.2025.1610560

**Published:** 2025-06-20

**Authors:** Lin Na, Jing Chang, Xinqi Li, Xiaona Che, Yunfei Sun, Wenjing Cui, Xin Xue

**Affiliations:** ^1^Department of Cardiology, The Second Hospital of Jilin University, Changchun, China; ^2^Clinical Laboratory, The Second Hospital of Jilin University, Changchun, China; ^3^Department of Cardiology, Xi'an International Medical Center Hospital, Xi'an, China

**Keywords:** cardiovascular disease, gut microbiota, dietary index, dietary pattern, NHANES

## Abstract

**Introduction:**

Cardiovascular disease (CVD) is a leading cause of mortality worldwide, and its development and progression are closely associated with diet-induced changes in gut microbiota. This study aims to investigate the relationship between the dietary index for gut microbiota (DI-GM) and its components, including the beneficial gut microbiota score (BGMS) and the unfavorable gut microbiota score (UGMS), in relation to cardiovascular disease.

**Methods:**

We conducted a cross-sectional study using National Health and Nutrition Examination Survey (NHANES) data (1999–2020), collecting baseline sociodemographic and health-related data from 41,193 adults aged ≥20 years. We constructed multivariable weighted logistic regression models to evaluate associations between DI-GM, BGMS, UGMS, and CVD risk, generating weighted restricted cubic spline (RCS) plots to visualize dose–response relationships. Subgroup analyses assessed outcome robustness across sex, age, hypertension, and diabetes subgroups.

**Results:**

After adjusting for confounders (age, sex, race, poverty-to-income ratio [PIR index], marital status, and education level, smoking status, alcohol consumption, body mass index [BMI], and comorbidities), significant associations emerged between DI-GM, BGMS, and CVD risk. Increasing DI-GM and BGMS levels showed gradually decreasing CVD risk trends (DI-GM: OR = 0.97, 95% CI: 0.94–1.00, *p* < 0.05; BGMS: OR = 0.92, 95% CI: 0.88–0.96, *p* < 0.05). No significant association was found between UGMS and CVD risk (OR = 1.04, 95% CI: 0.99–1.08, *p* > 0.05). Subgroup analyses revealed more significant DI-GM and BGMS associations with CVD risk in female versus male participants (*p* for interaction < 0.05).

**Conclusion:**

Among adults aged ≥20 years, DI-GM and BGMS showed significant inverse association with CVD risk. Compared to DI-GM, BGMS demonstrates a stronger inverse association with cardiovascular disease risk. These findings underscore the potential crucial role of favorable dietary patterns in cardiovascular disease prevention.

## 1 Introduction

Cardiovascular disease (CVD), including coronary artery disease, heart failure, stroke, and related conditions, exhibit a high incidence rate and represent the leading cause of death globally (1). Among modifiable risk factors—such as metabolic, environmental, and behavioral contributors—to cardiovascular disease burden, dietary risk ranks as the foremost behavioral risk factor (2, 3). Research demonstrates that diet critically influences the composition, function, and diversity of gut microbiota (4), which, along with their metabolites, significantly contribute to CVD progression (5).

The dietary index for gut microbiota (DI-GM) is a novel dietary index designed to assess gut microbiota health (6). It quantifies the influence of diet on gut microbiota composition and diversity by scoring 14 food or nutrient categories, including 10 beneficial and four detrimental components. A higher DI-GM score indicates a healthier gut microbiota. Unlike traditional dietary indices, DI-GM offers broader applicability and is better suited for personalized dietary guidance (7).

However, current understanding of the relationship between the gut microbiota dietary index and cardiovascular disease risk remains limited, necessitating further investigation. We will conduct a cross-sectional analysis using data from the National Health and Nutrition Examination Survey (NHANES) spanning 1999–2020 to examine associations between the gut microbiota dietary index, its components, and cardiovascular disease risk, aiming to provide personalized dietary strategies for the prevention of cardiovascular system diseases.

## 2 Materials and methods

### 2.1 Study population

This study conducted a cross-sectional analysis using data from the National Health and Nutrition Examination Survey (NHANES) from 1999 to 2020. NHANES is a nationally representative survey administered by the National Center for Health Statistics (NCHS) of the Centers for Disease Control and Prevention (CDC). It employs a complex, multistage probability sampling design to assess the health and nutritional status of the non-institutionalized U.S. population. The database is released biennially and includes health examination, laboratory test, and dietary interview data for participants of all ages. For this study, we included 107,622 participants from 11 NHANES cycles (1999–2020) as the source population. Exclusion criteria were: (1) Participants under 20 years of age. (2) Participants with missing data on cardiovascular disease (CVD) or dietary intake. The NHANES study protocol was approved by the NCHS Research Ethics Review Board, and all participants provided written informed consent. Detailed ethical review and consent documentation are available at: https://www.cdc.gov/nchs/nhanes/. This study complied with the Declaration of Helsinki and followed the STROBE guidelines for observational epidemiological studies. As the data were obtained from a publicly available database, the Ethics Review Committee of the Second Hospital of Jilin University waived additional ethical review.

### 2.2 Exposure variable

The Dietary Index of Gut Microbiota (DI-GM) serves as the independent variable in this study. Developed by Kase et al., the DI-GM is a scoring system designed to evaluate the influence of diet on gut microbiota health (6). The DI-GM incorporates both beneficial and detrimental dietary components. Beneficial components include avocado, broccoli, chickpeas, coffee, cranberries, fermented dairy products, fiber, green tea (excluded from scoring due to insufficient tea-type specificity in NHANES data), soy, and whole grains. Detrimental components comprise red meat, processed meats, refined grains, and high-fat diets (defined as ≥40% of energy from fat). The dietary component score is defined based on the median intake for a specific gender: for beneficial components, a score of 1 is assigned if the intake is ≥ the median, otherwise 0, summed up as the beneficial gut microbiota score (BGMS, range 0–10); for adverse components, a score of 0 is assigned if the intake is ≥ the median or 40% (high-fat diet), otherwise 1, summed up as the unbeneficial gut microbiota score (UGMS, range 0–4). The DI-GM score is the sum of all component scores, ranging from 0 to 14, with a higher score indicating a more favorable dietary pattern for gut microbiota health. However, in the NHANES database, since the 24-h dietary recall data does not record specific types of tea consumption, the DI-GM score in NHANES does not include the green tea component, and the DI-GM score ranges from 0 to 13.

### 2.3 Outcome variable

The outcome variable in this study was the occurrence of cardiovascular disease (CVD). CVD diagnosis was based on self-reported physician diagnoses obtained during individual interviews using a standardized medical conditions questionnaire. Participants were asked: “Has a doctor or other health professional ever told you that you have congestive heart failure, coronary heart disease, angina, myocardial infarction, or stroke?” Participants who answered “yes” to any of these conditions were classified as having CVD.

### 2.4 Evaluation of covariates

In this study, we collected baseline data on the social demographic status and health-related factors of the participants. The data were obtained through computer-assisted personal interviews (CAPI) conducted at the participants’ homes using a computer system. The collected data included information on age, gender, race, marital status, education, poverty-income ratio (PIR), body mass index (BMI), smoking status, drinking status, and the presence of hypertension, hyperlipidemia, and diabetes mellitus. Race/ethnicity was classified into five categories: non-Hispanic white, non-Hispanic black, Mexican-American, other Hispanic, and other races. Educational background was categorized based on the educational attainment of individuals aged 20 and above, including less than 9th grade, 9–11th grade (including 12th grade without a diploma), high school graduation/GED or equivalent, some college or an associate degree, and college graduate or higher. Marital status was classified into four categories: married, never married, living with a partner, and others (such as widowed, divorced, or separated). The PIR was calculated by dividing family income by the poverty line specific to family size and the corresponding year and state. The PIR was classified into low income (1.30), middle income (1.30–3.49), and high income (≥3.50). Smoking status was classified into three categories: never (less than 100 cigarettes), former (more than 100 cigarettes but quit smoking), and current (more than 100 cigarettes and currently smoking). Drinking status was classified as never drinking (less than 12 drinks in a lifetime), former drinking (more than 12 drinks in 1 year, no drinking last year, or no drinking last year but more than 12 drinks in a lifetime), and current drinking. The diagnosis of hypertension is established by meeting at least one of the following criteria: (1) a self-reported diagnosis from a physician, (2) the use of antihypertensive medications, or (3) an average systolic blood pressure of ≥140 mmHg or an average diastolic blood pressure of ≥90 mmHg. Similarly, the diagnosis of hyperlipidemia is determined by fulfilling at least one of the following criteria: (1) the use of lipid-lowering medications, (2) hypertriglyceridemia, defined as triglycerides ≥150 mg/dL, (3) hypercholesterolemia, characterized by total cholesterol ≥200 mg/dL, LDL ≥ 130 mg/dL, or HDL ≤ 40 mg/dL, (4) a fasting plasma glucose level of ≥7.0 mmol/L, (5) a random or 2-h oral glucose tolerance test plasma glucose level of ≥11.1 mmol/L, or (6) the use of diabetes medications or insulin. For comprehensive information regarding covariates, please refer to the NHANES website.^1^

### 2.5 Statistical analysis

In accordance with the NHANES database analysis guidelines (8, 9), we employed a complex, multistage probability sampling design and applied Mobile Examination Center (MEC) sample weights to ensure that the results were representative, unbiased, and provided precise estimates. The method for calculating sampling weights is as follows: For the periods 1999–2000 and 2001–2002, the weights are computed as WTMEC4YR multiplied by 2 divided by (11–1 + 1.625). For the period 2003–2016, the weights are computed as WTMEC2YR divided by (11–1 + 1.625). Finally, for 2017–2020, the weights are calculated as WTMECPRP multiplied by 1.625 and then divided by (11–1 + 1.625).

We categorized the respondents into two groups based on the presence of cardiovascular disease: Group 1, the non-cardiovascular disease group (No-CVD), and Group 2, the cardiovascular disease group (CVD). Quantitative data were expressed as weighted means (mean ± standard error) and analyzed using the Wilcoxon rank-sum test for complex survey samples to assess differences between variables. Categorical data were presented as counts (n) and weighted percentages (%), and analyzed using the Rao-Scott chi-square test.

A weighted logistic regression model was utilized to evaluate the associations between DI-GM, BGMS, and UGMS with the risk of cardiovascular disease (CVD). Results were reported as 95% confidence intervals (CI) and odds ratios (OR). Three models were constructed to adjust for potential confounding factors. Model 1 adjusted only for age; Model 2 additionally accounted for sex, race, poverty-to-income ratio (PIR index), marital status, and education level; Model 3 further included smoking status, alcohol consumption, body mass index (BMI), and comorbidities (hypertension, hyperlipidemia, and diabetes). Notably, when examining the association between BGMS (or UGMS) and CVD risk, UGMS (or BGMS) was also incorporated as a covariate. All regression analyses considered the survey’s sample weights and employed tolerance values and variance inflation factor (VIF) statistics to assess multicollinearity among variables. A *p*-value threshold of less than 0.05 was adopted to determine statistical significance.

Weighted restricted cubic spline (RCS) plots were constructed to illustrate the dose–response relationships between DI-GM, BGMS, UGMS, and the risk of cardiovascular disease. Restricted cubic spline plots with three knots were established at the 10th, 50th, and 90th percentiles of baseline DI-GM, BGMS, and UGMS, respectively. The reference points for these analyses were set as the medians of DI-GM, BGMS, and UGMS.

To evaluate the robustness of the association, pre-specified weighted subgroup analyses were conducted. Stratified analyses were carried out separately by age, sex, presence of hypertension, and diabetes mellitus to examine the associations between DI-GM, BGMS, and the risk of cardiovascular disease. The likelihood ratio test was employed to assess interactions among subgroups.

Since the population in this cross-sectional study was derived from a large database with a substantial sample size, sample size determination is rarely required in such research. However, to assess statistical power, we performed a *post hoc* calculation using PASS 2023 (NCSS statistical software) to evaluate the associations. According to the Global Burden of Disease (GBD) study,^2^ the prevalence (p) of cardiovascular disease among adults aged ≥20 years in the United States is approximately 0.17. With a two-tailed significance level (*α*) of 0.05, an allowable error (*δ*) of 0.017 (0.1 times the prevalence), and a two-tailed 95% confidence interval width of 0.034 (2 times the allowable error), the minimum required sample size was 1,993 individuals. All analyses were performed using Free software (version 2.1) and R statistical software (version 4.2.2; R Foundation for Statistical Computing, Vienna, Austria). A two-sided *p*-value <0.05 was considered statistically significant.

## 3 Results

### 3.1 Participant selection

**Figure 1:**
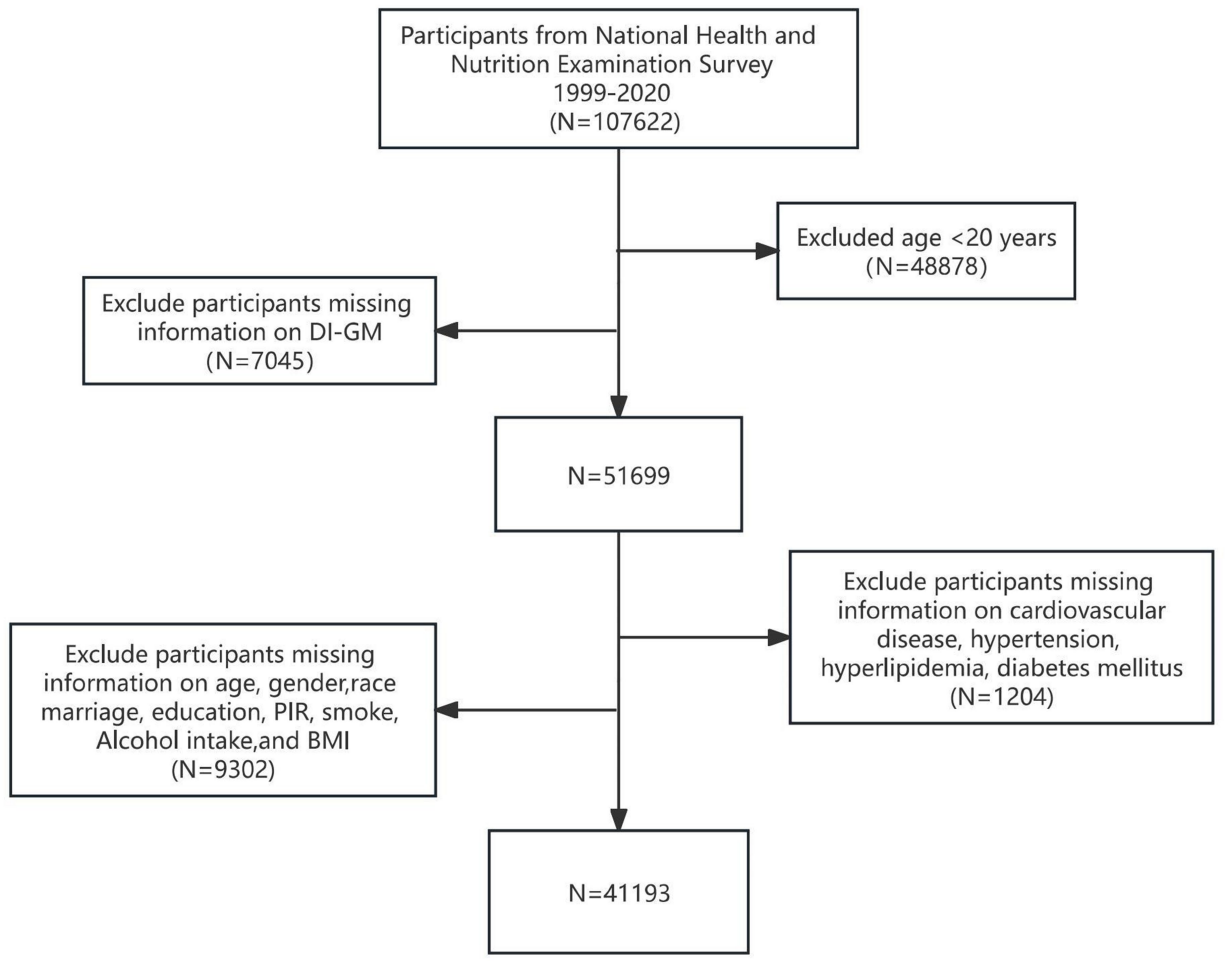
The flowchart of participants selection.

We included a total of 107,622 participants from the NHANES study data, which spans from 1999 to 2020. Among these, 48,878 participants were excluded due to being under the age of 20 years, while 7,045 were excluded for missing data on dietary intake and general metabolism (DI-GM). Furthermore, 9,032 participants were excluded due to incomplete information regarding age, race, marital or educational status, poverty income ratio (PIR), smoking habits, alcohol consumption, or body mass index (BMI). Additionally, 1,204 participants were excluded owing to missing data on CVD and comorbidities, such as hypertension, diabetes, and hyperlipidemia. Ultimately, 41,193 participants who met the inclusion criteria were included as study subjects. Figure 1 illustrates the participant selection flowchart.

### 3.2 The weighted baseline characteristics of the participants

Table 1 summarizes the weighted baseline clinical characteristics of the study population stratified by the presence or absence of CVD. A total of 41,193 participants were included, of whom 20,688 were male (49.2%) and 4,591 had CVD (11.1%). The mean age was 47.00 (0.20) years, and the mean DI-GM score was 4.53 (0.02). Compared with individuals without CVD, those with CVD tended to be older, had a higher proportion of males, more smokers, higher BMI levels, and a greater prevalence of hyperlipidemia, hypertension, and diabetes. Additionally, patients with CVD exhibited lower BGMS scores and higher UGMS scores (*p* < 0.05).

**Table 1:** Weighted baseline clinical characteristics of participants in the CVD group and No-CVD group.

Characteristic	Overall, *N*^1^=156,805,661 (*n*^2^ = 41,193)	No-CVD, *N* = 143,234,017 (*n* = 36,602)	CVD, *N* = 13,571,644 (*n* = 4,591)	*p*-value
Age, mean (SE)	47.00 (0.20)	45.37 (0.19)	64.19 (0.29)	<0.001
Gender, *n* (%)				<0.001
Male	20,688 (49.25%)	18,040 (48.80%)	2,648 (54.04%)	
Female	20,505 (50.75%)	18,562 (51.20%)	1,943 (45.96%)	
Race, *n* (%)				<0.001
Non-Hispanic White	19,138 (70.62%)	16,500 (69.95%)	2,638 (77.68%)	
Non-Hispanic Black	8,628 (10.39%)	7,669 (10.38%)	959 (10.51%)	
Mexican American	6,761 (7.54%)	6,268 (7.90%)	493 (3.74%)	
Other Hispanic	3,243 (5.24%)	2,965 (5.43%)	278 (3.29%)	
Other Race	3,423 (6.20%)	3,200 (6.34%)	223 (4.79%)	
Marry, *n* (%)				0.011
Married/Living with partner	24,801 (64.13%)	22,189 (64.35%)	2,612 (61.83%)	
Never married/Other	16,392 (35.87%)	14,413 (35.65%)	1,979 (38.17%)	
PIR group, *n* (%)				<0.001
Low income	12,258 (20.21%)	10,604 (19.54%)	1,654 (27.37%)	
Middle income	15,644 (35.58%)	13,768 (35.07%)	1,876 (40.96%)	
High income	13,291 (44.21%)	12,230 (45.40%)	1,061 (31.67%)	
Education, *n* (%)				<0.001
Less than 9th grade	4,321 (4.97%)	3,595 (4.56%)	726 (9.31%)	
9–11th Grade	5,822 (10.51%)	5,014 (10.06%)	808 (15.28%)	
High School Grad/GED or Equivalent	9,590 (24.02%)	8,431 (23.66%)	1,159 (27.91%)	
Some college or AA degree	12,133 (31.50%)	10,904 (31.69%)	1,229 (29.50%)	
College graduate or above	9,327 (28.99%)	8,658 (30.04%)	669 (18.00%)	
Smoke, *n* (%)				<0.001
Never	22,044 (53.59%)	20,280 (55.05%)	1,764 (38.13%)	
Former	10,413 (25.23%)	8,530 (23.81%)	1,883 (40.19%)	
Now	8,736 (21.19%)	7,792 (21.14%)	944 (21.68%)	
Alcohol intake, *n* (%)				<0.001
Never	5,552 (10.69%)	4,907 (10.52%)	645 (12.47%)	
Former	6,902 (13.56%)	5,457 (12.19%)	1,445 (28.05%)	
Current	28,739 (75.75%)	26,238 (77.29%)	2,501 (59.48%)	
BMI (kg·m^2^), mean (SE)	28.88 (0.06)	28.73 (0.07)	30.50 (0.14)	28.88 (0.06)
Hyperlipidemia, *n* (%)				<0.001
No	12,441 (31.01%)	11,778 (32.75%)	663 (12.70%)	
Yes	28,752 (68.99%)	24,824 (67.25%)	3,928 (87.30%)	
Hypertension, *n* (%)				<0.001
No	23,584 (62.79%)	22,549 (66.26%)	1,035 (26.15%)	
Yes	17,609 (37.21%)	14,053 (33.74%)	3,556 (73.85%)	
DM, *n* (%)				<0.001
No	34,104 (87.28%)	31,313 (89.42%)	2,791 (64.60%)	
Yes	7,089 (12.72%)	5,289 (10.58%)	1,800 (35.40%)	
DI-GM, mean (SE)	4.53 (0.02)	4.53 (0.02)	4.57 (0.03)	0.046
BGMS, mean (SE)	2.22 (0.01)	2.23 (0.02)	2.17 (0.03)	0.047
UGMS, mean (SE)	2.31 (0.01)	2.30 (0.01)	2.40 (0.02)	<0.001

1 N represents weighted counts to reflect the population distribution, while ^2^n represents unweighted counts from the actual sample size. DI-GM, dietary index for gut microbiota; BGMS, beneficial to gut microbiota score; UGMS, unfavorable to gut microbiota score; BMI, body mass index; DM, diabetes mellitus; PIR, poverty income ratio; SE, standard error.

### 3.3 The association between DI-GM, BGMS, and UGMS and the risk of CVD

To evaluate whether DI-GM and its components are independently associated with the risk of CVD in adult participants aged 20 years or older, we constructed a multivariate weighted logistic regression model to examine their relationship (Table 2). Table 2 displays the results of the multivariate weighted regression analysis for DI-GM, BGMS, UGMS, and CVD. A multivariate adjustment model was utilized to confirm the robustness of the findings. In Model 1, only age was adjusted for. In Model 2, adjustments were made for age, sex, race, PIR index, marital status, and education level. In Model 3, further adjustments were included for smoking status, alcohol consumption, BMI, and comorbidities (hypertension, hyperlipidemia, diabetes). The results demonstrated that, after accounting for confounding factors, there was an association between DI-GM, BGMS, and the risk of cardiovascular disease. As the levels of DI-GM and BGMS increased, the risk of CVD exhibited a gradually decreasing trend. Specifically, for every 1-unit increase in DI-GM, the risk of cardiovascular disease decreased by 3% (OR = 0.97, 95% CI: 0.94–1.00, *p* < 0.05). For every 1-unit increase in BGMS, the risk of cardiovascular disease decreases by 8% (OR = 0.92, 95% CI: 0.88–0.96, *p* < 0.001). UGMS is not associated with the risk of cardiovascular disease (OR = 1.04, 95% CI: 0.99–1.08, *p* > 0.05).

**Table 2:** Weighted multivariate regression analysis of the association between DI-GM, BGMS, UGMS, and cardiovascular disease risk.

Variables	Model 1 OR (95%CI)	*p*-value	Model 2 OR (95%CI)	*p*-value	Model 3 OR (95%CI)	*p*-value
DI-GM	0.89 (0.87, 0.92)	<0.001	0.94 (0.91, 0.96)	<0.001	0.97 (0.94, 1.00)	0.040
BGMS	0.85 (0.82, 0.88)	<0.001	0.90 (0.87, 0.94)	<0.001	0.92 (0.88, 0.96)	<0.001
UGMS	0.98 (0.94, 1.02)	0.378	1 (0.96, 1.04)	0.953	1.04 (0.99, 1.08)	0.103

DI-GM, dietary index for gut microbiota; BGMS, beneficial to gut microbiota score; BMI, body mass index; UGMS, unfavorable to gut microbiota score; OR, odds ratio; CI, confidence interval.

Model 1 was adjusted for age.

Model 2 was adjusted for age, gender, race, PIR, marital status, and education.

Model 3 extended Model 2 by smoking status, alcohol consumption, body mass index (BMI), hypertension, hyperlipidemia, and diabetes. UGMS was also included as a covariate when analyzing BGMS in Model 3 and BGMS was also included as a covariate when analyzing UGMS in Model 3.

**Figure 2:**
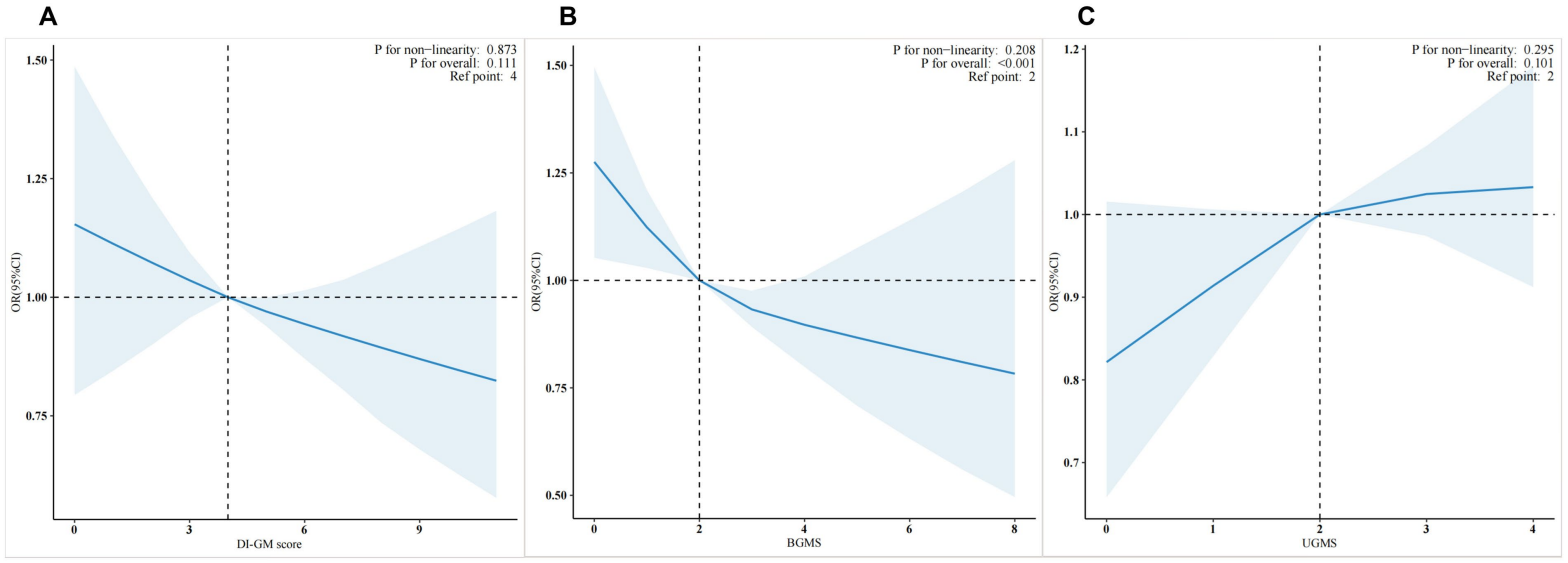
Weighted restricted cubic spline plots of DI-GM **(A)**, BGMS **(B)**, and UGMS **(C)** in relation to cardiovascular disease risk”

To evaluate the dose–response relationships between DI-GM, BGMS, and cardiovascular disease risk after adjustment using Model 3, weighted restricted cubic spline plots with three knots were constructed at the 10th, 50th, and 90th percentiles of the baseline DI-GM, BGMS, and UGMS indices (Figure 2). The reference points were set as the medians of DI-GM, BGMS, and UGMS. As shown in the figure, the risk (incidence) of cardiovascular disease demonstrated a decreasing trend with increasing BGMS levels (*p* for overall < 0.05). However, for DI-GM and UGMS, the dose–response relationship with cardiovascular disease risk is not significant (*p* for overall >0.05).

### 3.4 Subgroup analysis

**Figure 3:**
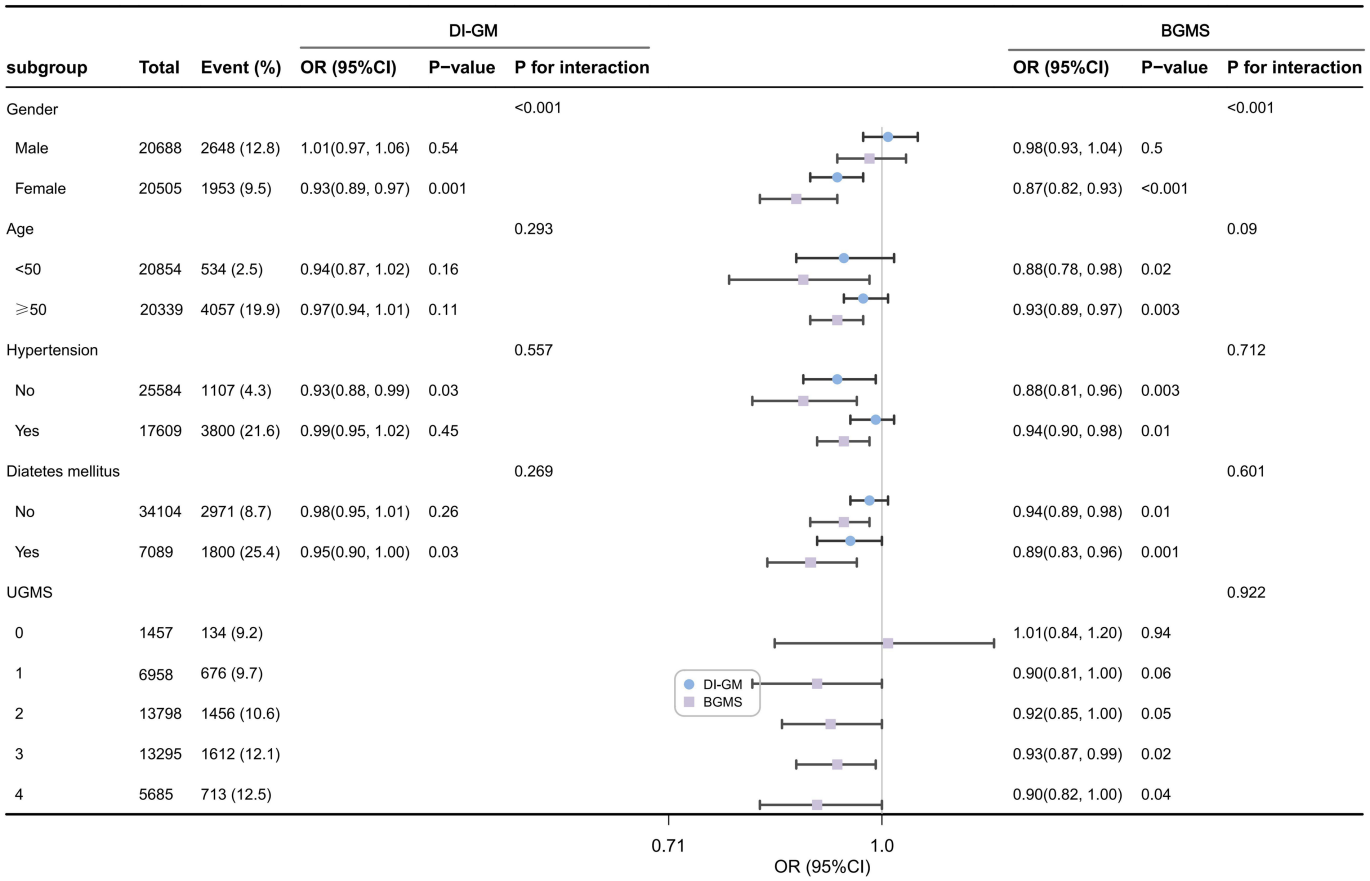
Forest plot of weighted subgroup analysis of the relationship between DI-GM, BGMS, and cardiovascular disease risk.

Figure 3 presents the forest plot of the weighted subgroup analysis examining the relationship between DI-GM, BGMS, and cardiovascular disease (CVD) risk. The subgroup analysis results indicate that, after adjusting for confounding factors, a significant interaction exists between gender and DI-GM or BGMS in gender-stratified patient subgroups (*p* for interaction < 0.001). Compared to males, the association between DI-GM and CVD risk was more pronounced in female patients (OR = 0.93, 95% CI: 0.89–0.97, *p* < 0.001). Similarly, the association between BGMS and CVD risk was also stronger in female patients (OR = 0.87, 95% CI: 0.82–0.93, *p* < 0.001). In subgroups stratified by age (<50 years vs. ≥50 years), hypertension status, and diabetes status, the associations of DI-GM and BGMS with CVD risk were comparable (*p* for interaction > 0.05). Additionally, the subgroup analysis revealed that for patients with a UGMS score of ≤2, the association between BGMS and CVD risk was not statistically significant (*p* > 0.05).

## 4 Discussion

### 4.1 Summary of research findings

In this cross-sectional study, we observed that the risk of cardiovascular disease progressively decreased with increasing levels of the Dietary Index of Gut Microbiota (DI-GM) and its component BGMS. Notably, BGMS exhibited a stronger protective effect against cardiovascular disease compared to DI-GM. After adjusting for potential confounding factors, including age, sex, ethnicity, PIR index, marital status, education level, smoking status, alcohol consumption, BMI, and comorbidities (hypertension, hyperlipidemia, diabetes), using multivariable weighted logistic regression analysis, this association remained statistically significant. These findings suggest that higher levels of DI-GM and BGMS may be independently associated with a reduced risk of cardiovascular disease. However, no statistically significant association was observed between UGMS, another component of DI-GM, and the risk of cardiovascular disease. Furthermore, subgroup analyses revealed that the inverse relationship between DI-GM, BGMS, and cardiovascular disease risk was more pronounced in the female population. Additionally, in the subgroup with UGMS scores ≤ 2, which indicates excessive intake of dietary components unfavorable to gut microbiota, the protective effect of BGMS on cardiovascular disease was not evident (*p* > 0.05).

### 4.2 Comparison with previous studies

Previous studies investigating the association between gut microbiota, diet, and CVD have encompassed various dietary patterns, including those that are beneficial or detrimental to gut microbiota. Regarding diets beneficial to gut microbiota, such as those high in dietary fiber, numerous scholars have identified their positive effects on cardiovascular health. A systematic review and meta-analysis conducted by Threapleton et al., which analyzed 22 prospective studies, revealed an inverse correlation between total dietary fiber intake and the risk of CVD (10, 11). Research by Sonnenburg et al. further demonstrated that healthy high-fiber dietary patterns, such as the consumption of fruits, vegetables, and whole grains, may have preventive effects against CVD (12). Epidemiological studies led by Joanne Slavin and others have also shown that adequate dietary fiber intake can reduce cardiovascular disease risk by lowering low-density lipoprotein cholesterol levels (13). Our findings align with these prior conclusions, indicating that the intake of gut microbiota-beneficial diets (e.g., dietary fiber) plays a critical role in the prevention of CVD.

Similarly, a systematic review by Tom Butler and colleagues on dietary patterns improving cardiovascular disease outcomes highlighted that the Mediterranean diet—characterized by vegetables, fruits, nuts, legumes, unrefined grains, moderate amounts of fish and shellfish, and fermented dairy products—can significantly reduce all-cause mortality from cardiovascular disease (14). BGMS, the key focus of our research, comprises seven components consistent with the Mediterranean diet standards, further supporting its protective effects on cardiovascular health. Additionally, our study found that compared to DI-GM, BGMS exhibits a stronger association with the risk of CVD, suggesting that the protective effects of DI-GM on the cardiovascular system may primarily stem from its beneficial components. Existing evidence underscores that a diet rich in vegetables, fruits, whole grains, legumes, nuts, seeds, low-fat dairy products, lean protein, and foods low in saturated fats, added sugars, and sodium is widely recognized as a healthy dietary pattern (15–17). Such dietary components beneficial to gut microbiota fall within the scope of a healthy diet and demonstrate protective effects against CVD (15–17). These findings collectively highlight the importance of dietary patterns in mitigating cardiovascular disease risk.

Conversely, consuming an unhealthy diet high in saturated fats increases susceptibility to cardiovascular diseases (12). Another prospective cohort study by Victor W. Zhong et al. found that higher intake of processed meats and unprocessed red meats was significantly associated with an increased risk of CVD events among U.S. adults (18). A 6-month randomized controlled feeding trial conducted by Chinese scholar Yi Wan demonstrated that a high-fat diet (HFD) may induce gut dysbiosis, impair the intestinal barrier, and ultimately elevate the risk of CVD (19). In our study, the association between UGMS and the risk of cardiovascular system diseases was not statistically significant. This discrepancy may arise from the scoring method of UGMS, which is calculated based on gender-specific median intakes rather than absolute thresholds of intake, potentially masking the consumption levels of high-risk diets. Since UGMS is a relative value assessment, its scoring may vary dynamically when applied to diverse populations worldwide. Therefore, more research is needed to explore the absolute thresholds of gut-microbiota-unfavorable dietary intake associated with cardiovascular disease risk. We will conduct further investigations in future studies by integrating additional public databases and self-established databases. Although our study did not reveal a correlation between UGMS and the risk of cardiovascular diseases, subgroup analysis results indicated that when the intake of diets unfavorable to gut microbiota is excessive (i.e., UGMS ≤ 2), the protective effects of gut microbiota-beneficial diets cannot be fully exerted on cardiovascular system diseases. This suggests that excessive consumption of diets unfavorable to gut microbiota, such as high-fat diets, may have adverse effects on the cardiovascular system, thereby masking the protective effects of BGMS diets on the cardiovascular system. Therefore, to achieve the protective effects of BGMS on the cardiovascular system, it may be necessary to control the intake of red meat, processed meat, refined grains, and high-fat diets (i.e., UGMS > 2). Recently, a cross-sectional study by Liu et al., utilizing the NHANES database for adults aged over 30, demonstrated a linear negative correlation between DI-GM, BGMS, and the prevalence of stroke, while the correlation between UGMS and stroke was not significant (20). This finding aligns with our results. As stroke is one of the cardiovascular system diseases, this study further supports our research conclusions.

### 4.3 The association mechanism between DI-GM and cardiovascular disease risk

Regarding the association between DI-GM and cardiovascular disease risk, a complex underlying mechanism exists (34). The gut microbiota may play a critical role in the occurrence, progression, and treatment of CVD through various pathways. Firstly, the gut microbiota metabolizes dietary components (such as choline, carnitine, and dietary fiber) to produce a variety of metabolites that directly or indirectly influence the cardiovascular system. For diets beneficial to gut microbiota (e.g., whole grains, legumes, and fiber), short-chain fatty acids generated via gut microbiota fermentation can reduce the risk of CVD, such as hypertension, by modulating immune responses, suppressing inflammation, and improving vascular endothelial function (21, 22). Conversely, for diets detrimental to gut microbiota (e.g., red meat and high-fat diets), reducing their intake can inhibit the synthesis of trimethylamine N-oxide (TMAO), thereby exerting anti-atherosclerotic effects (23). Additionally, beneficial dietary components, such as dietary fiber and probiotics, can enhance microbial diversity, alter bile acid metabolism to maintain cholesterol homeostasis, and suppress inflammatory responses, thus protecting the cardiovascular system (24–26). Furthermore, a high-fiber diet can increase the thickness of the mucus layer, upregulate the expression of tight junction proteins, reduce intestinal permeability, protect intestinal barrier function, and prevent microbial metabolites (e.g., TMAO and endotoxins) from entering the circulatory system, thereby mitigating the risk of cardiovascular damage (27–29).

### 4.4 Gender differences in DI-GM and its components in relation to cardiovascular risk

Our study also revealed that the associations between DI-GM, BGMS, and the risk of CVD are more pronounced in the female population, suggesting that a diet beneficial to gut microbiota provides stronger cardiovascular protection for women. This may be attributed to gender differences in gut microbiota composition. Salvado et al. found that women exhibit higher microbiome diversity (30) and a greater abundance of potentially protective gut microbiota genera (30). Research by Renzo et al. demonstrated that in the female population, administration of a Mediterranean diet led to upregulation of the APOE and ACE genes, which are beneficial for regulating blood lipid and blood pressure levels (31). Moreover, estrogen has been shown to enhance intestinal barrier function by regulating the synthesis and secretion of intestinal mucins (32). Consequently, the structure of the female gut microbiota may be more responsive to dietary fibers and similar diets, potentially leading to the production of more beneficial metabolites. Additionally, compared to males, females tend to adhere better to healthy diets, with higher intake of dietary fibers and lower consumption of high-energy diets such as fats (33).

### 4.5 The significance for clinical practice

The findings of this study advocate for the incorporation of gut microbiota-targeted dietary strategies into cardiovascular disease (CVD) prevention efforts. Public health recommendations should emphasize the consumption of gut-friendly diets, such as whole grains and dietary fibers, while judiciously limiting the intake of foods detrimental to gut microbiota, including processed meats, high-fat foods, and other pro-inflammatory options. It is important to note that gender differences may exist in gut microbiota composition. Therefore, precise individualized dietary interventions tailored to different genders may enhance preventive efficacy. In clinical practice, the DI-GM index offers clinicians an innovative approach to assess the gut microbiota health status of patients.

### 4.6 Limitations

This study has several limitations. First, due to its cross-sectional design, the lack of time-series data precludes the establishment of causal relationships, necessitating further longitudinal or prospective studies. However, this study employed a large-scale, nationally representative NHANES cohort and performed weighted analyses to minimize confounding bias, thereby accurately reflecting the health status of the U.S. population. The stratified analyses suggest that the findings remain robust across different subgroups. Second, the DI-GM score captures participants’ dietary habits at the time of data collection. Nevertheless, unless influenced by health conditions, most adults maintain relatively stable dietary patterns. Additionally, since the NHANES 24-h dietary recall data did not record specific tea types, green tea components were not included in the DI-GM score, which may reduce the validity of BGMS. In subsequent studies, we will incorporate other databases (containing green tea data) beyond NHANES to further explore the association between DI-GM, its components, and cardiovascular disease risk. Furthermore, reliance on self-reported dietary recall may introduce measurement bias. In future studies, we will incorporate metagenomic sequencing and improve repeated dietary assessment data to further clarify the findings. Finally, since our *post hoc* power analysis was based on the overall CVD prevalence in adults aged ≥20 years, the subgroup analyses may have been underpowered.

## 5 Conclusion

In summary, our study reveals a significant inverse association between the Dietary Index for Gut Microbiota (DI-GM) and the Beneficial Gut Microbiota Score (BGMS) with the risk of cardiovascular diseases among adults aged 20 and above. Compared to DI-GM, BGMS demonstrates a stronger inverse association with cardiovascular disease risk. These findings underscore the potential critical role of adhering to a healthy dietary pattern in the prevention of cardiovascular diseases.

## Data Availability

Publicly available datasets were analyzed in this study. This data can be found at: https://wwwn.cdc.gov/nchs/nhanes/.

## Footnotes

^1^ https://www.cdc.gov/nchs/nhanes/index.htm

^2^ https://vizhub.healthdata.org/gbd-results/
